# *Pa*VarDB: a database and web platform for missense variant analysis in *Pseudomonas aeruginosa* using an end-to-end *BVbase* pipeline

**DOI:** 10.1093/database/baag014

**Published:** 2026-03-24

**Authors:** Virudhagiri Elamurugan, Subbiah Thamotharan, Vigneshwar Ramakrishnan

**Affiliations:** DBT-Bioinformatics Center, School of Chemical & Biotechnology, SASTRA Deemed University, Tirumalaisamudram, Thanjavur, Tamilnadu, India - 613401; DBT-Bioinformatics Center, School of Chemical & Biotechnology, SASTRA Deemed University, Tirumalaisamudram, Thanjavur, Tamilnadu, India - 613401; Biomolecular Crystallography Lab, School of Chemical & Biotechnology, SASTRA Deemed University, Tirumalaisamudram, Thanjavur, Tamilnadu, India - 613401; DBT-Bioinformatics Center, School of Chemical & Biotechnology, SASTRA Deemed University, Tirumalaisamudram, Thanjavur, Tamilnadu, India - 613401; Computational Molecular Biophysics Lab, School of Chemical & Biotechnology, SASTRA Deemed University, Tirumalaisamudram, Thanjavur, Tamilnadu, India - 613401

## Abstract

Genomic variant data are useful in detecting and treating antibiotic-resistant bacteria. However, there are no bacterial genomic variant databases that catalogue the variations in the different genes across strains. In this work a Nextflow- and Docker-based end-to-end pipeline, *BVbase*, that can automate the creation of databases from raw high-throughput sequences has been created to fill this lacuna with *Pseudomonas aeruginosa* as a case study. *Pseudomonas aeruginosa* is a Gram-negative adaptable pathogen with multiple antibiotic resistances that causes various types of infections, including respiratory, urinary, and bloodstream infections. The pipeline can take multistrain genomic files, detect missense variants, and save results in a database with the help of Python and SQLite (https://github.com/bic-sastra/BVbase). Using the generated database for *P. aeruginosa*, a web application interface has been made using Flask and HTML that runs in a server with MySQL backend (https://bic.sastra.edu/pavardb). The web application provides supports for different types of queries to select variants by gene, geographical group, isolation country, antibiotics, and resistance phenotype. This web interface generates results as variant tables, plots, and statistics for the selected data. By enabling interactive visualizations and advanced selection, the platform supports research and clinical use through the exploration of genomic variations associated with antimicrobial resistance.

## Introduction

Antimicrobial resistance (AMR) is one of the most serious global health challenges today. The World Health Organization has estimated that, if the current trends continue, AMR could result in 10 million deaths per year by 2050 [1]. Among the bacteria driving this crisis, *Pseudomonas aeruginosa* is especially worrisome because it is naturally resistant to many antibiotics and can easily acquire new resistance mechanisms [2]. This Gram-negative pathogen is a major cause of hospital-acquired infections, such as pneumonia, bloodstream infections, urinary tract infections, and wound infections, particularly affecting patients with weakened immune systems or chronic conditions like cystic fibrosis.

*Pseudomonas aeruginosa* uses multiple strategies to resist antibiotics, including reduced outer membrane permeability, production of β-lactamases, and activation of drug-efflux systems such as MexAB-OprM [2]. These resistance mechanisms make treatment even more challenging. Additionally, the bacterium can form biofilms, which shield it from both antibiotics and the immune system [3]. As a result, infections caused by *P. aeruginosa* are often severe, leading to longer hospital stays, and significantly higher healthcare costs [4].

Nonsynonymous single nucleotide polymorphisms (nsSNPs) are genetic changes that result in changes in the amino acid sequence of proteins and can affect their function. In *P. aeruginosa*, these kinds of mutations are closely linked to antibiotic resistance. For example, mutations in the *gyrA* and *parC* genes are known to drive resistance to fluoroquinolones, while alterations in the *oprD* gene can reduce the uptake of carbapenems, making them less effective [2]. Another example is the overproduction of the AmpC β-lactamase enzyme, usually because of mutations in regulatory genes like *ampD* or *ampR*, which makes the bacterium resistant to extended-spectrum cephalosporins [5]. Thus, detecting these mutations can help guide treatment decisions. A repository of such mutations over a period of time can also shed light on the evolution mechanism of resistance development. Whole-genome sequencing methods, combined with variant annotation tools such as SnpEff, now make it faster and easier to identify these nsSNPs and understand their role in antibiotic resistance [6].

Some pipelines like BACTOPIA [7], BPGA [8], MicroPIPE [9], BAGEP [10], BacSeq [11], and bacLIFE [12] have been developed to make tasks such as bacterial genome assembly, variant calling, and annotation more efficient. These tools produce standard outputs like VCF, FASTA, and GFF files. In other words, the functionality of these tools ends at the data generation stage. None of those tools lead to a web-based interface that helps researchers, particularly for those in clinical and translational settings, to interact with and search through. For *P. aeruginosa*, a pathogen of high clinical importance due to its resistance to multiple antibiotics, there is a notable absence of a focused database that catalogues key gene mutations associated with drug resistance. Bridging this gap is quintessential to explore and compare genetic mutations between different clinical strains, correlate them with resistance phenotype, and identify marker mutations for more targeted treatments.

In this work, we develop a fully automated workflow that can, not only identify missense variants from the input data, but also store the information in a structured database. Using a Nextflow framework, the work integrates the entire genomic analysis process from quality checking, trimming to alignment, variant calling, annotation, and filtering. This is followed by processing the output as a structured database that can be appended as new data arrives. We anticipate that such a workflow can be easily deployed in a clinical setting, enabling clinical researchers to create databases from their own clinical samples. As a case study, we have used this pipeline for cataloguing the nonsynonymous mutations in *P. aeruginosa* strains from publicly available database. This workflow also integrates the metadata (including details such as resistance phenotype, geographical location, and isolation country). This facilitates correlating the mutations in different genes to the antibiotic resistance profiles and can be used as the basis for understanding their role in resistance. The final output is a comprehensive, web-searchable database built with Python, SQLite3, and Flask, which will aid in the development of targeted therapies and clinical studies. The pipeline, which can be used for any bacteria of interest, is also made available publicly so that it can aid in the creation of local databases at clinical research institutes.

In the next section, we briefly describe the materials and methods, including the data source for the database and the processing steps. This is then followed by the results and discussion, where we discuss the statistics of the data, database schema, and some analysis from the database.

## Materials and methods

### Input data

The assembled genomes (FNA files) of *P. aeruginosa* were obtained from the Bacterial and Viral Bioinformatics Resource (BV-BRC) database [13]. A total of 2342 genomes were included in this study, 1561 strains from Asia and 781 strains from other regions. The dataset filtering was based on the Taxon ID 287 with complete metadata. The metadata file, which includes information such as genome ID, SRA accession id, strain name, isolation country, geographical group, resistant phenotype, antibiotic, and taxon ID was also obtained. The genome IDs of these genomes are unique identifiers for each strain and were used to ascertain that the genomes used in the study did not have any duplicates. *Pseudomonas aeruginosa* PAO1 strain was used as the reference genome (FASTA file). These three files (assembled genomes, reference genome, and metadata) are used as inputs to our pipeline. The genome IDs of the 2342 genomes and the reference genome are available in the Supplementary Material S1. Our pipeline can also handle raw reads (single-end and paired-end) instead of assembled genomes.

### End-to-end pipeline for variant analysis

**Figure 1:**
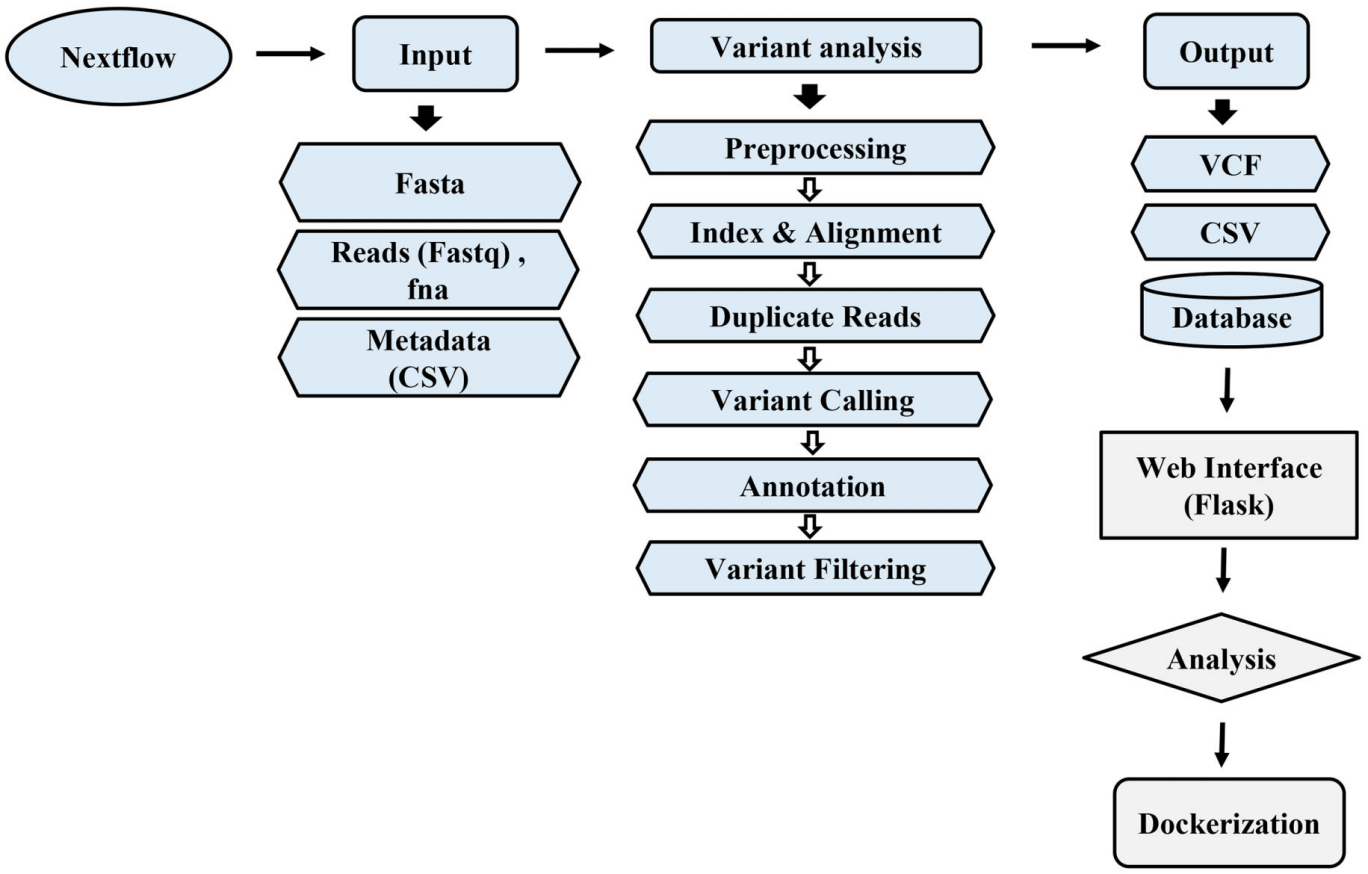
Overview of the variant analysis and database implementation workflow for *P. aeruginosa*.

*BVbase*, our dockerized nextflow-based pipeline [14] (supported for both Ubuntu and Windows platforms) for variant analysis (https://github.com/bic-sastra/BVbase) was then used to identify the SNPs. Fig. 1 captures the sequence of steps in the identification of SNPs and database creation as used in the pipeline. We provide a brief description of the steps involved below.

#### Indexing and preprocessing

The process starts with indexing the reference genome for efficient alignment. Faidx in samtools was used to index the reference genome (FASTA file) [15]. This is followed by the below steps:

- **Alignment:** The assembled genomes are aligned to the indexed reference genome using BWA-MEM (Burrows-Wheeler Aligner - Maximal Exact Matches) [16] method. This step typically requires around 6 GB of RAM.
- **SAM to BAM Conversion:** The alignment output in SAM format is converted into the binary BAM format using samtools.
- **Sorting:** The BAM files are then sorted by genomic coordinates using samtools sort option
**Duplicate Removal:** PCR duplicates, which can introduce bias in variant calling, are removed using the samtools markdup option. The default parameters for samtools markdup were used, which include distance setting of 100 bp and template mode.

#### Variant Calling

Bcftools [17] was used to identify the variants, including SNPs and indels. This step requires around 6 GB of RAM. The output is a VCF (variant call format) [18] file containing the identified variants with quality and depth details of the variants.

#### Annotation and missense variant extraction

Before annotation, the column corresponding to the chromosome name in the VCF file is renamed as 1, as this was necessary for SnpEff [19] annotation. SnpEff was then used to annotate the variants with their biological information. Instead of the in-built reference database for *P. aeruginosa* PAO1 available within SnpEff (bin file), a custom SnpEff database was created for comprehensive annotation. This includes the integration of the reference GFF, the genomic FASTA file, and the in-built bin file along with the cds.fa and protein.fa files created using Prokka [20]. The detailed process is available in the manual of our pipeline. This annotation process also requires around 6 GB of RAM. Our pipeline can also filter out the low-quality variants. This step is then followed by the extraction of missense variants from the annotated VCF file.

#### Processing for database generation

The extracted file containing only the missense variants is then processed so that it can be used for database generation. This includes chromosome name replacement using sed, converting the VCF file into CSV format, including the genome IDs from the metadata, and including a separate column of amino acid changes. This processed output CSV file and the metadata file are then used to generate an SQLite database using Python script that is integrated with the pipeline and is available on Github. This script also supports appending data to an existing database. Key fields such as country, geographic group, phenotype, antibiotic, and gene names are indexed to allow efficient querying.

### Web interface

A user-friendly web interface was built using Flask (HTML) for server hosting. The application is currently available at https://bic.sastra.edu/pavardb, specifically designed for *P. aeruginosa* variant data. This platform supports data visualization through tables, charts, and statistical summaries. Users can filter results based on phenotype or region with some fields marked as mandatory. The results of the user searches can also be downloaded as a CSV file.

## Results and discussion

### Data summary

**Figure 2:**
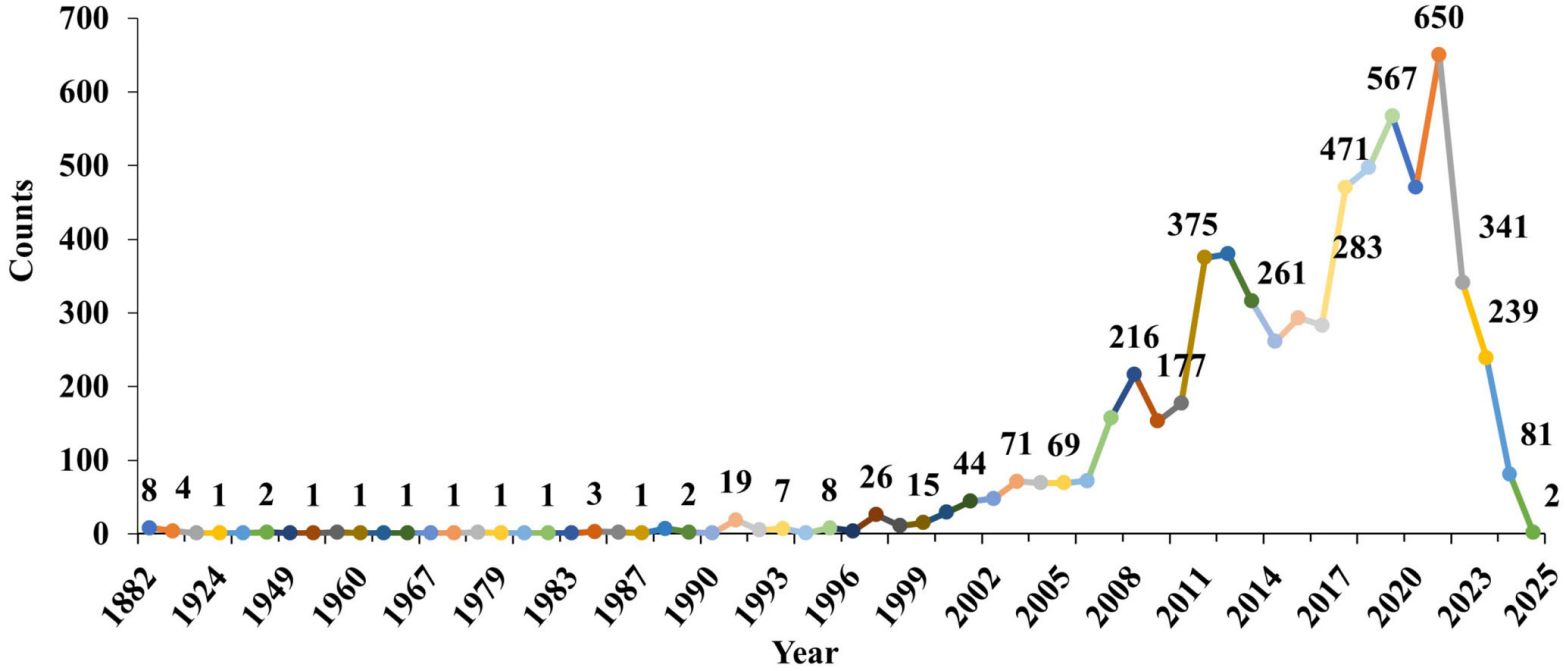
Year-wise counts of *P. aeruginosa* genomes retrieved from BV-BRC (Bacterial and Viral Bioinformatics Resource Center).

**Figure 3:**
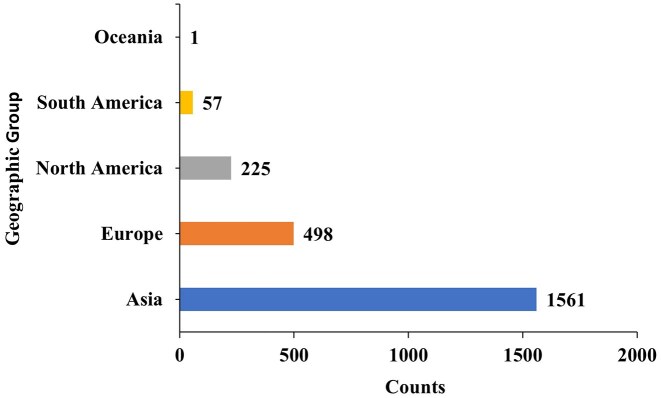
The count of *P. aeruginosa* genomes from geographic group shows that Asia (1561) and Europe (498) report the largest number of isolates, followed by North America (225), South America (57), and Oceania [1].

**Figure 4:**
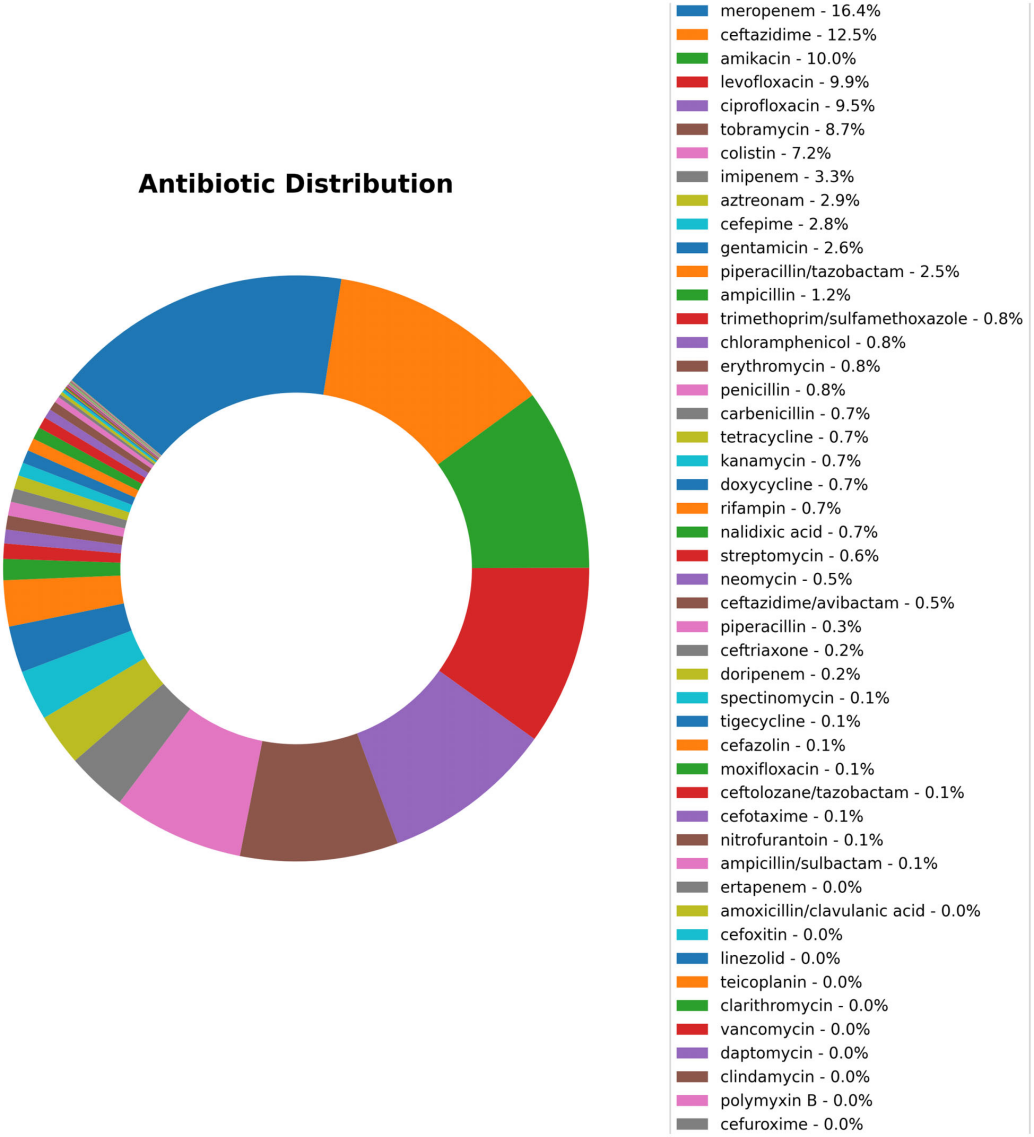
The proportion of genomes resistant to the different antibiotics in the 2342 genomes of *P. aeruginosa* shows that meropenem constitutes the highest percentage of resistance cases (16.4%), followed by ceftazidime (12.5%), amikacin (10.0%), and levofloxacin, which represents 9.9%.

We begin with a brief overview of the data in the database. The year-wise counts of the genomes in the database are given in Fig. 2. The maximum number of genomes is from the year 2021. The geographic group contributing to the maximum number of genomes is Asia (1561), followed by Europe (498), as seen in Fig. 3. In total, 2342 genomes were included in this study; 1561 strains from Asia and 781 strains from other regions. The dataset was filtered based on the taxon ID 287 with complete metadata. Thus, strains from several regions with incomplete metadata were not included, and thus the dataset may not fully represent the global distribution of *P. aeruginosa*. The data contains 4720 resistant phenotypes and 5004 susceptible phenotypes and 740 intermediate phenotypes (across all antibiotics) among the 2342 genomes. The proportion of genomes resistant to the various antibiotics is shown in Fig. 4. It is observed that the majority of the genomes (16.4%) are resistant to meropenem.

### Database generation and visualization

**Figure 5:**
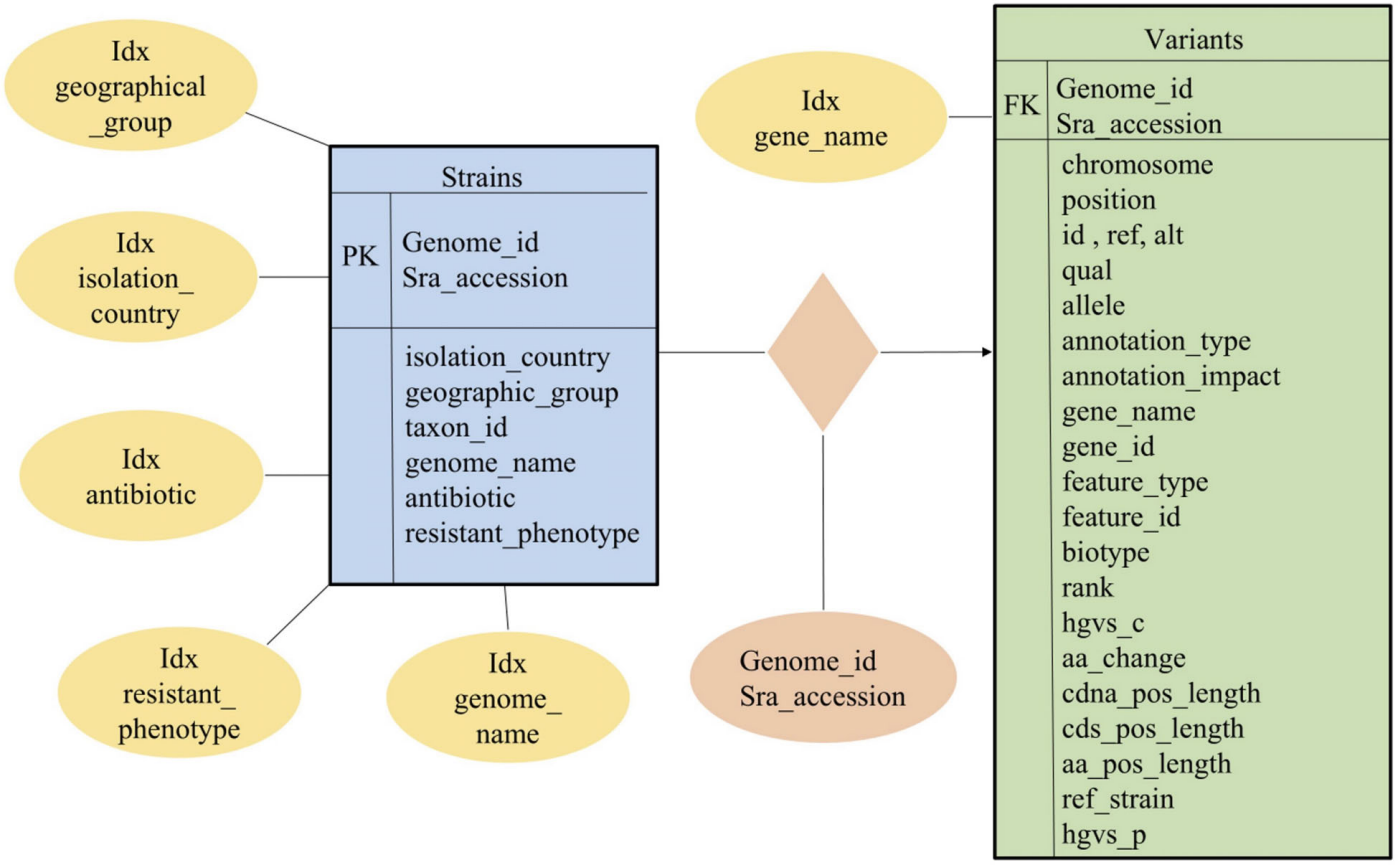
*Pa*VarDB is organized using a relational database schema, implemented in SQLite.

The 2342 genomes were then processed using our pipeline. This resulted in a total of 20 011 846 missense variants. All the processed variant data (VCF files) were converted to structured CSV files and imported into an SQLite database. The database schema for hosting the server included fields such as genome ID, gene name, mutation type, amino acid change, phenotype, and associated antibiotic (Fig. 5). For hosting on the server, the data was imported into MySQL through CSV files generated from the SQLite database. Metadata, including isolation source and geographic location, were integrated to support epidemiological studies. The pipeline was configured with 6 GB of RAM allocated to annotation, alignment, and variant calling processes to ensure efficient execution. The total runtime for processing these genomes in this pipeline, including database generation, is ∼8 h 30 min in a system with a configuration of Intel Core i7 14th Gen processor and 32 GB of RAM. The total data processed is around 10.5 GB.

The database schema (Fig. 5) comprises two primary tables: Strains (represented in blue) and Variants (shown in green), which are connected via Genome_id and Sra_accession. The Strains Table contains metadata that includes details such as the isolation country, geographic group, taxon ID, genome name, antibiotic utilized, and resistance phenotype. The Variants Table contains variant information, positional data, annotation, and amino acid changes. To enhance efficient querying and analysis, indexation (illustrated as yellow ellipses) was done on crucial searchable fields such as gene name, antibiotic, and resistant phenotype.

**Figure 6:**
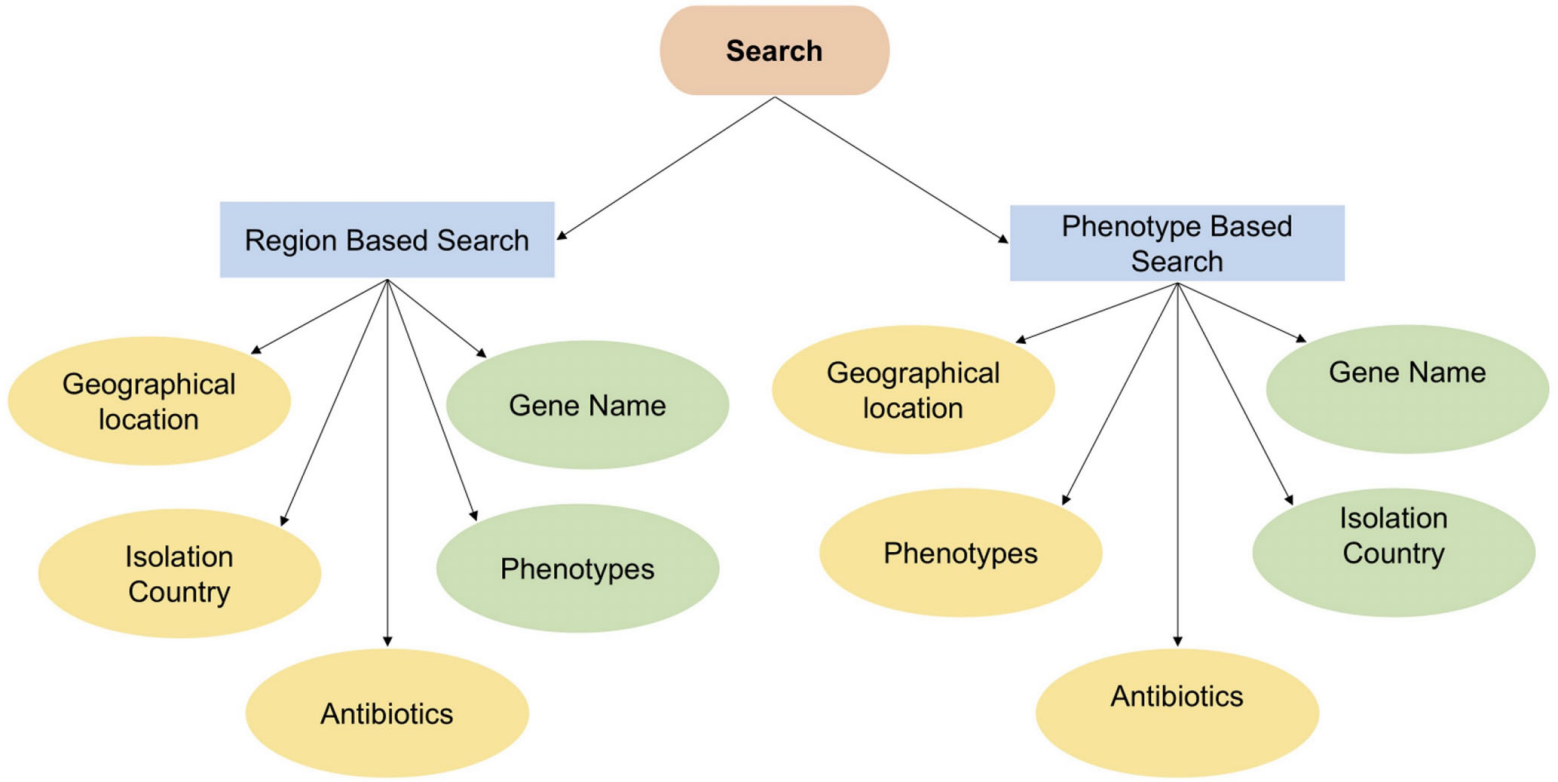
The search schema for the web-based interface of *Pa*VarDB (*P. aeruginosa* variant database) is organized into two primary categories: Region-based search and phenotype-based search.

The web interface (*Pa*VarDB), developed using Flask (HTML), gives the users the option to explore the database either via phenotype-based or region-based filtering (Fig. 6). The region-based search enables users to apply filters based on mandatory (yellow) criteria of geographical location, country of isolation, and optional (green) criteria such as gene name and phenotypes. Phenotype-based search, on the other hand, allows users to filter using optional (green) criteria like gene name and country of isolation, alongside mandatory (yellow) aspects such as antibiotics, phenotypes, and geographical origin. These two ways of searching give flexibility to the users for analysis and visualization of resistance-related variants. Visual outputs include tables, plots, and summary statistics. Thus, this platform offers an accessible way for researchers to analyse variant data.

### Frequently mutated genes

To illustrate the usefulness of the database, we identify the top three genes that have the greatest number of mutations by querying the database. Bcftools [17] was used to identify and filter the variants. Table 1 shows the top three genes with the number of variations observed and their function.

**Table 1:** Top three frequently mutated genes and their functional role in *P. aeruginosa*

S. No.	Gene name	Length of the protein (no. of amino acids)	Unique variant count	Total variant count	No. of genomes in which gene is present	Function
1	PA1874	2468	2213	127 721	2318	Hypothetical protein (predicted ABC transporter)
2	PA0041	3535	2004	85 458	2310	Filamentous haemagglutinin FhaB/tRNA nuclease CdiA-like TPS
3	PA2462	5627	1872	86 440	1768	Filamentous haemagglutinin FhaB/tRNA nuclease CdiA-like TPS

The PA1874 is part of the PA1874-77 operon that likely codes for an ABC transporter [21]. While the molecular function of PA1874 is not yet completely elucidated, it has been implicated in biofilm formation and multidrug resistance in *P. aeruginosa* [21]. The high sequence diversity observed in this gene is indicative of the evolutionary pressure it is under. Analysis of mutation frequency reveals a major hotspot at amino acid position 1145. Variations at this specific position were identified in 2127 genomes (Supplementary Material S2). While position 1145 shows the highest *frequency* of change, amino acid position 2174 exhibits the highest *complexity* of variation (Supplementary Material S3). This position accommodates six distinct types of mutations, identifying it as a hypervariable site capable of tolerating a wide range of amino acid substitutions without compromising protein stability. The presence of distinct hypervariable regions (positions 1145 and 2174) is consistent with the characteristics of large surface-exposed proteins, and the variability at position 2174 likely facilitates the structural adaptation of the transporter’s extracellular domain, potentially altering substrate specificity or modifying surface properties to enhance biofilm resilience in diverse clinical environments.

PA0041 (*cdiA1*) and PA2462 (*cdiA2*) encode the filamentous exoproteins of Contact-Dependent Growth Inhibition (CDI) systems, which facilitate interbacterial competition via toxin delivery and contribute to host-pathogen interactions and virulence [22, 23]. Our observation of the high sequence diversity of these genes aligns with the literature on the diversity of the C-terminal domain of CDI, which is thought to help drive diversity within the species [22] (Supplementary Material S3–S7).

While the above analysis is restricted to the three genes that have the maximum number of mutations, our database facilitates such analysis, giving insights into variations in genes/proteins across genomes. Such insights can be very useful in drug target identification, drug design, and vaccine development.

We also analysed the mutation rate across the entire genomes of the 2342 *P. aeruginosa* isolates (provided in Supplementary Material S8). The data reveals a median mutation rate of ∼0.69% and a mean mutation rate of 0.76%. This finding is consistent with existing literature, which reports the conserved nature of the *P. aeruginosa* core genome as having a sequence diversity of ∼0.5%–0.7% [24]. We also identified four specific genomes that exhibit significantly higher mutation rates than the rest (Table 2). Strains with elevated mutation frequencies—often termed ‘hypermutators’—have been frequently reported in clinical isolates, particularly those from chronic cystic fibrosis infections [25].

**Table 2:** Genomes with high mutation rate

Genome ID	Mutation rate
287.9905	6.89%
287.7805	4.52%
287.12724	4.51%
287.34357	4.5%

While the background genomic mutation rate is ∼0.7%, the identified top three genes (PA0041, PA2462, and PA1874) exhibit mutation rates between 33% and 89%. This represents approximately two orders of magnitude higher mutation than the rest of the genome (which has a median of 0.69%). Given the functional roles of these genes discussed above, it is expected that they evolve at accelerated rates to adapt to environmental pressures. Taken together, this database serves as a vital reference for identifying hotspot positions in genes as well as the genes driving the evolutionary dynamics of *P. aeruginosa*.

## Conclusion

The creation of the variant analysis pipeline, *BVbase*, has facilitated the creation of a comprehensive missense variant database for *P. aeruginosa* from genomic data. This pipeline supports both Windows and Ubuntu environments, and portability via Docker. The availability of a GUI also makes the pipeline easily accessible for non-bioinformatics researchers. This pipeline is now available for public use (https://github.com/bic-sastra/BVbase). In this work, we have used *BVbase* to process 2342 *P. aeruginosa* assembled genomes collected from across the world. This resulted in generating >20 011 846 variant data. The database generated from the pipeline has been interfaced with a web (*Pa*VarDB) for search and analysis purposes, and it is available for public use (https://bic.sastra.edu/pavardb/). This interface provides options to search variants according to user needs and generates output data as tables, charts, and summary statistics. The analysis of the database can form the basis for several applications, including drug target identification, drug design, vaccine development, and relating the variants to specific phenotypes, etc., thus paving the way for improving diagnostic methods and therapeutic strategies.

## Supplementary Material

baag014_Supplemental_Files

## Data Availability

BVbase Tool Respository : https://github.com/bic-sastra/BVbase. PaVarDB Web Platform: https://bic.sastra.edu/pavardb/.
